# Electroacupuncture Inhibits the Activity of Astrocytes in Spinal Cord in Rats with Visceral Hypersensitivity by Inhibiting P2Y_1_ Receptor-Mediated MAPK/ERK Signaling Pathway

**DOI:** 10.1155/2020/4956179

**Published:** 2020-02-25

**Authors:** Jingming Zhao, Hui Li, Chong Shi, Tiezheng Yang, Baoshi Xu

**Affiliations:** ^1^Proctology Department, Affiliated Hospital of Changchun University of Chinese Medicine, Changchun 130021, China; ^2^Geriatric Department, Affiliated Hospital of Changchun University of Chinese Medicine, Changchun 130021, China

## Abstract

**Background:**

Irritable bowel syndrome (IBS) is a chronic functional bowel disease characterized by abdominal pain and changes in bowel habits in the absence of organic disease. Electroacupuncture (EA) has been shown to alleviate visceral hypersensitivity (VH) in IBS rat models by inhibiting the activation of astrocytes in the spinal cord. However, the underlying molecular mechanisms mediated by P2Y_1_ receptor of this effect of electroacupuncture remain unclear.

**Aim:**

To explore whether EA inhibits the activity of astrocytes in the spinal cord dorsal horn of rat with visceral hypersensitivity by inhibiting P2Y_1_ receptor and its downstream mitogen activated protein kinase/extracellular regulated kinase 1 (MAPK/ERK) pathway.

**Methods:**

Ten-day-old Sprague-Dawley (SD) male rats were given an intracolonic injection of 0.2 ml of 0.5% acetic acid (AA) to establish a visceral hypersensitivity model. EA was performed at Zusanli (ST 36) and Shangjuxu (ST 37) at 100 Hz for 1.05 s and 2 Hz for 2.85 s alternately, pulse width for 0.1 ms, 1 mA, 30 min/d, once a day, for 1 week. Cytokines IL-6, IL-1*β*, and TNF-*α* were analyzed by ELISA. The expressions of the P2Y_1_ receptor and pERK1/2 were analyzed by Western Blot and real-time PCR in the model and EA treated animals to explore the molecular mechanism of EA in inhibiting the activity of spinal cord dorsal horn (L_6_-S_2_ segment) astrocytes in rats with IBS visceral hypersensitivity.

**Results:**

EA significantly reduced the behavioral abdominal withdrawal reflex score (AWRs) of IBS rats with visceral hypersensitivity induced by AA. For comparison, intrathecal injection of astrocytes activity inhibitor fluorocitrate (FCA) also reduced visceral hypersensitivity in IBS rats. EA at Zusanli and Shangjuxu inhibited the mRNA and protein expression of the glial fibrillary acidic protein (GFAP) and in rat spinal cord and reduced the release of inflammatory cytokines IL-6, IL-1, and TNF-*α* were analyzed by ELISA. The expressions of the P2Y_1_ receptor and pERK1/2 were analyzed by Western Blot and real-time PCR in the model and EA treated animals to explore the molecular mechanism of EA in inhibiting the activity of spinal cord dorsal horn (L_6_-S_2_ segment) astrocytes in rats with IBS visceral hypersensitivity. *β*, and TNF-*μ*g, 10 *μ*g, 10

**Conclusion:**

EA inhibited astrocyte activity in the spinal cord dorsal horn of rat with IBS visceral hypersensitivity by inhibiting the P2Y_1_ receptor and its downstream, PKC, and MAPK/ERK1/2 pathways.

## 1. Introduction

IBS is a chronic functional bowel disease characterized by abdominal pain and changes in bowel habits without an organic disease [1]. Visceral hypersensitivity is believed to be a key underlying mechanism that causes pain. A recent systematic review and meta-analysis of randomized controlled trials (RCT) of clinical acupuncture for IBS confirmed the effectiveness of traditional Chinese medicine acupuncture for IBS [2–5]. Electroacupuncture (EA) can inhibit visceral injury responses at multiple levels [6, 7]. EA at body surface acupoints can inhibit visceral injury response at multiple nerve levels with the largest inhibitory effect on spinal dorsal horn [8]. EA can also significantly inhibit the expression of NMDA receptor 1 (NR1) [9] and NR2B receptors [10, 11] in the spinal dorsal horn in relieving visceral hypersensitivity [12, 13]. In recent years, it has been found that activation of spinal glial cells is a key factor in the occurrence and persistence of visceral hypersensitivity [14]. Activation of mature astrocytes is mainly manifested as enhanced GFAP expression which is necessary for the completion of gliosis of reactive astrocytes. Intestinal inflammation injury leads to the activation of spinal cord astrocytes which release a large number of neuroactive substances, enhance the sensitivity and reactivity of dorsal horn neurons, and generate central sensitization [15, 16]. For example, 2,4,6-trinitrobenzene sulfonic acid (TNBS) can lead to the enhancement of long-term potentiation (LTP) in pain synaptic transmission of the spinal dorsal horn in colitis rats [17]. The purinergic system regulates the activity of the glutamate energy system [18] which affects the NMDA receptor and also plays an important role in the communication of glial cells by regulating glutamate release in glial cells of the spinal cord [19, 20]. Clinical studies have found that the expression of P2Y_1_ and P2Y_2_ receptors in rectal sigmoid mucosa of patients with IBS-D was increased and that P2Y_2_ was associated with abdominal pain [21]. Activation of the P2Y receptor stimulates visceral hypersensitivity receptors in mice and humans highlighting the role of the P2Y-dependent mechanism in visceral hypersensitivity in gastrointestinal diseases [22]. P2Y_1_ receptor is an important pharmacological target for regulating the excitability of human colon smooth muscle which provides the basis for the development of peripheral target drugs for the relief of abdominal pain [23, 24]. EA has been shown to inhibit glial cell activation and lumbar spinal cord [25, 26] in the visceral hypersensitivity rat model induced by colon-rectum distention which provides the morphological basis for the inhibition of glial cells by EA; however, the molecular mechanisms remain unclear. Burnstock proposed the novel hypothesis for the involvement of purinergic signaling in acupuncture [27, 28]. There are many studies on purine receptor family mediated acupuncture analgesia [29, 30]. Protein kinase C (PKC) is an important signaling molecule of pain transmission and plays an important role in the central sensitization of visceral inflammatory pain [31]. The translocation of PKC to the cell membrane plays a role in mediating pain after visceral inflammatory pain stimulation [31]. EA stimulation can significantly inhibit the PKC membrane translocation level thereby inhibiting the pain signal transmission process and thus playing an analgesic role in the visceral hypersensitivity [32]. Upon P2Y_1_ receptor activation, the endogenous glutamate released by astrocytes has an excitatory regulatory effect on neurons [33]. In this study, we tested the hypothesis that EA may inhibit the activity of glial cells by jointly inhibiting the P2Y_1_ receptor-mediated activation of PKC and MAPK/ERK pathway to relieve visceral hypersensitivity.

## 2. Materials and Methods

### 2.1. Animals

Newborn male Sprague-Dawley rats, ten-day-old pups, were obtained from the Animal Center of Changchun University of Chinese Medicine with approval from the Animal Research Ethics Committee (number of the permit: SCXK- (Ji) 2018-0006). Rats were housed with a 12-hour alternating light-dark cycle (light on 7:00 A.M.) in a temperature- and humidity-controlled room (22 ± 2°C, 50–55%) and were provided with free access to food and water. All experimental procedures were performed in accordance with the guidelines of The International Association for the Study of Pain.

### 2.2. Induction of Visceral Hypersensitivity

The experimental animal model of IBS with visceral hypersensitivity was induced by neonatal colonic inflammation as previously described [34]. In brief, ten-day-old pups were administered an infusion of 0.2 ml of 0.5% acetic acid solution in saline into the colon 2 cm from the anus, only once. Control rats received an equal volume of normal saline. EA treatments were performed in the seventh week.

### 2.3. Electroacupuncture and Drugs Treatment

EA was applied at Zusanli (ST 36) and Shangjuxu (ST 37) acupoints. The Zusanli acupoint (ST36) is 3-4 mm below the knee joint and 1-2 mm outside the tibia crest. Shangjuxu acupoint (ST37) is located 7-8 mm below the knee joint and 1-2 mm outside the tibia crest [35]. The rat was fixed in a self-made fixator. Pair of stainless needles were inserted bilaterally at a depth of 5 mm. The EA treatment was given once a day from day 43 to day 49 using an EA apparatus (G-6805-2, China) with a constant rectangular current of alternating trains of dense-sparse frequencies. The stimulus parameters [36] were as follows: 100 Hz for 1.05 s, 2 Hz for 2.85 s alternately, pulse width for 0.1 ms, 1 mA, 30 min/d, once a day. Each group of experimental sample size is introduced in the results section. FCA, ADP, MRS, and SCH were applied by intrathecal injection. We anesthetized the rats with continued 1.5% isoflurane during the procedure. A 25 *μ*l Hamilton syringe attached to a 32G needle was carefully inserted between the groove of L_5_ and L_6_ vertebrae and observed for a tail flick as this sign indicates a successful entry of the needle in the intradural space. We secured the needle position with one hand and injected the desired volume of drugs with the other hand slowly. The injection frequency was once a day.

### 2.4. Behavioral Visceromotor Response to Colorectal Distension (CRD)

Visceromotor response (VMR) to CRD was commonly used as an objective outcome in the behavioral evaluation of abdominal pain in rats [37]. We adopted the AWRs for quantitative analysis as previously reported [38]. Briefly, the CRD stimulation was performed using a self-made balloon stimulator connected to a desktop sphygmomanometer and a syringe through a three-way valve to provide the distension stimulation at a constant pressure. When the rat was awake, the balloon was inserted from the anus along the rectum to reach the descending colon of the rat after the treatment of EA. CRD pain stimulation was applied at four different levels of pressure: 20, 40, 60, and 80 mmHg. Three AWR scores were measured for each rat with each measurement lasting approximately 20 s and with 3 min interval between measurements. The mean value was calculated as the final score. The detailed criteria are as follows [39].

### 2.5. ELISA

To quantitate concentration of IL-6, IL-1*β*, and TNF-*α* in spinal cord tissues, L_6_-S_2_ segments of spinal cord were cut and placed into 1.5 mL Eppendorf tubes and spun at 10000 ×g for 10 minutes, then placed in a new 1.5 mL Eppendorf tube, and used as samples (1/10 and 1/100 (v/v) dilutions). Cytokines IL-6, IL-1*β*, and TNF-*α* were performed using the rat ELISA Kit (eBioscience). Absorbance was read at 450 nm and concentrations of each were calculated with a standard curve. Concentrations were normalized to spinal cord weight.

### 2.6. Real-Time PCR

The total RNA of the spinal cord (L_6_-S_2_) was extracted using Qiagen RNeasy mini kit (Qiagen, Valencia, CA). PCR conditions were set as follows: amplifications 95°C for 30 s and thermal cycling 40 cycles at 95°C for 10 s and 60°C for 45 s. The following sequences were used for the primers for P2Y_1_ F:5′-CCTGCCTGCGGTCTACATCTTA-3′. P2Y_1_ primer R:5′-ACACCGTCAGGACAATTATCACCA-3′. GAPDH primer F:5′-GGCAAGTTCAACGGCACAGT-3′. GAPDH primer R:5′-ATGACATACTCAGCACCGGC-3′. ERK1/2 primer F:5′- CTCAAGCCTTCCAACCTC-3′. ERK1/2 primer R:5′ -TTCCACGGCACCTTATTT-3′. PKC primer F:5′-CCTGCCTGCGGTCTACATCTTA-3′. PKC primer R: 5′-ACACCGTCAGGACAATTATCACCA-3′. Fold change difference in gene expression was calculated with 2^−ΔΔ*Ct*^ method using GAPDH as a housekeeping gene.

### 2.7. Western Blot

Colon-specific (L_6_-S_2_) spinal cord samples were homogenized in lysis buffer (Invitrogen). Protein concentration was determined by BCA Protein Assay Kit (Invitrogen). Protein samples were separated on instant SDS–PAGE gel and transferred to PVDF membranes. Blots were blocked with 5% milk and incubated overnight at 4°C with an antibody against GFAP (1 : 500, abcam), P2Y_1_ (1 : 500, abcam), pERK1/2 (1 : 200, R&D Systems), and PKC (1 : 300, R&D Systems). The anti-rabbit IgG concentration of goat was 1 : 1000. The blots were further incubated with anti-rabbit IgG secondary antibodies for 2 h at room temperature and captured by Imaging Lab. The intensity of the bands was quantified using ImageJ (NIH, Bethesda, MD).

### 2.8. Statistical Analysis

SPSS21.0 was used for statistical analysis of the data. Data represent mean ± SEM. The difference between the groups was analyzed by one-way ANOVA or Kruskal-Wallis H test or nonparametric test. Multiple comparisons between means of multiple samples were performed using the Nemenyi method, and *P* < 0.05 was considered statistically significant.

## 3. Result

### 3.1. The Effect of FCA and Electroacupuncture on Visceral Hypersensitivity in Acetic Acid-Induced IBS Rat Model

**Figure 1:**
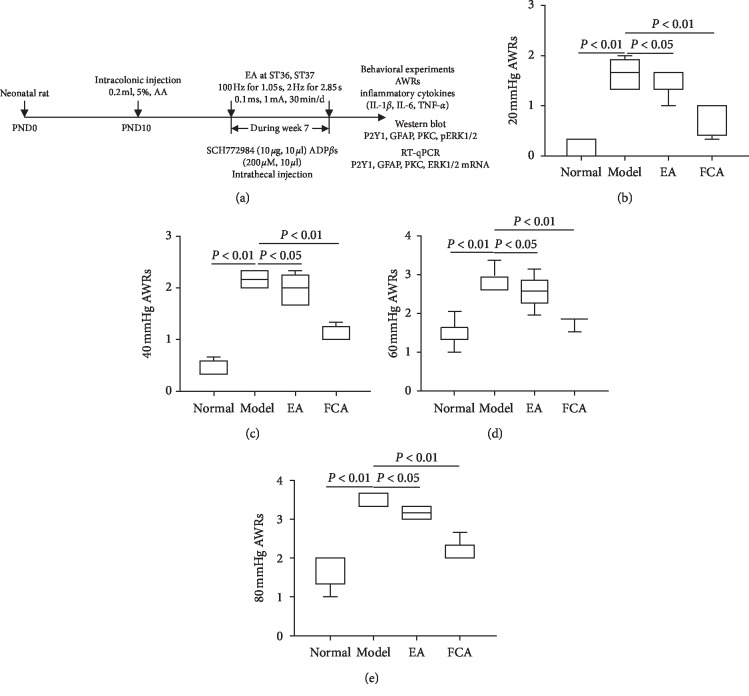
Experimental flow chart and the inhibitory effect of FCA on visceral hypersensitivity. (a) Experimental flow chart: neonatal SD rats were administered an intracolonic dose of 5% acetic acid for induction of visceral hypersensitivity of IBS. EA and drug intervention were conducted at the beginning of week 7 and behavioral scores and molecular detection were conducted at the end of week 7. (b)–(e) AWRs of visceral hypersensitivity under stimulation of 20, 40, 60, and 80 mmHg CRD. Normal (*n* = 8), Model (*n* = 8), EA (*n* = 8), and FCA (*n* = 8).

The detailed experimental procedure of this study is shown in Figure 1(a), which is described as follows. A 2 cm catheter was inserted into the anus of 10-day-old male rats and 0.2 ml of 0.5% acetic acid solution was introduced by intracolonic injection. Both EA stimulation and intrathecal injection were conducted at the beginning of week 7, and behavioral scoring, ELISA, and protein and mRNA indexes were analyzed at the end of week 7. The AWR score of the Model group rats was higher than that of normal control group rats under four different levels of pressure CRD, behavior scoring criteria referred to Table 1 [39]: 20 mmHg (*P* < 0.01, Figure 1(b)), 40 mmHg (*P* < 0.01, Figure 1(c)), 60 mmHg (*P* < 0.01, Figure 1(d)), and 80 mmHg (*P* < 0.01, Figure 1(e)). These results suggest that acetic acid induced visceral hypersensitivity in normal rats which lasted into adulthood (7 weeks). The behavioral characteristics of visceral hypersensitivity in experimental animals were comparable to those in IBS patients. Compared with Model group, EA at Zusanli (ST 36) and Shangjuxu (ST 37) (100 Hz for 1.05 s, 2 Hz for 2.85 s alternately, pulse width for 0.1 ms, 1 mA, 30 min/d, once a day) significantly reduced the behavioral AWR scores of IBS rats at 20 mmHg (*P* < 0.05, Figure 1(b)), 40 mmHg (*P* < 0.05, Figure 1(c)), 60 mmHg (*P* < 0.05, Figure 1(d)), and 80 mmHg (*P* < 0.05, Figure 1(e)), suggesting that EA significantly inhibited chronic visceral hypersensitivity in rats with acetic acid-induced IBS model. Previous studies have shown that chemical inflammatory injury of the colon caused peripheral and central sensitization and neurons [40] show increased excitability and glial cells were involved in the occurrence and maintenance of sensitization [41]. In order to verify whether astrocytes in the spinal cord were also involved in spinal sensitization in visceral hypersensitivity in our model, we conducted intrathecal injection of fluorocitrate (FCA), an inhibitor of glial cell activity, to observe the effect of FCA on AWR score of the rat with visceral hypersensitivity. Results showed that the AWR score of the FCA group rats was significantly lower than that of the Model group under the stimulation of CRD, 20 mmHg (*P* < 0.01, Figure 1(b)), 40 mmHg (*P* < 0.01, Figure 1(c)), 60 mmHg (*P* < 0.01, Figure 1(d)), and 80 mmHg (*P* < 0.05, Figure 1(e)), indicating that inhibiting activation of astrocyte reduced chronic visceral hypersensitivity in IBS model which is consistent with previous reports on the role of glial cells in other inflammatory visceral hypersensitivities [17].

### 3.2. Regulation of GFAP and P2Y_1_ Receptor by Electroacupuncture in the Spinal Cord of Rat with Visceral Hypersensitivity

EA can effectively inhibit the behavioral score of adult rats with visceral hypersensitivity induced by mechanical CRD stimulation [25] and in somatic pain models such as neuropathic pain and inflammatory pain. EA can also exert an inhibitory effect on hyperalgesia by inhibiting the activity of astrocytes in the spinal dorsal horn [26, 42, 43]. As the inhibitory effect of EA at Zusanli (ST 36) and Shangjuxu (ST 37) acupoints on chronic inflammatory visceral hypersensitivity induced by acetic acid was similar to that of FCA, we speculated that EA may play a role in alleviating visceral hypersensitivity by inhibiting the activity of glial cells. First, we tested the regulatory effect of EA on GFAP, the astrocyte marker, and protein and mRNA expression in the spinal cord of rats with visceral hypersensitivity as well and on the levels of cytokines (IL-6, IL-1*β*, and TNF-*α*).

**Figure 2:**
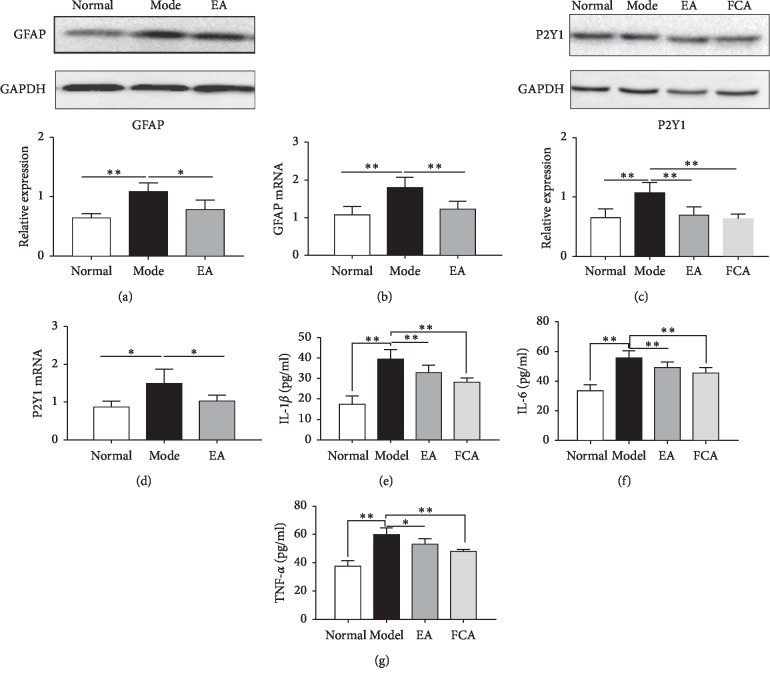
Regulation of GFAP and P2Y_1_ receptor by electroacupuncture in the spinal cord of rat with visceral hypersensitivity. (a) The expression of GFAP protein in the L_6_-S_2_ spinal dorsal segments, *n* = 8. (b) Expression of GFAP mRNA in L_6_-S_2_ spinal cord segments, *n* = 8. (c) Expression of P2Y_1_ in spinal dorsal, *n* = 8. (d) P2Y_1_ mRNA expression of spinal dorsal, *n* = 8. (e)–(g) The concentrations of IL-6, IL-1*β*, and TNF-*α* in the spinal cord, *n* = 8. ^*∗*^*P* < 0.05, ^*∗∗*^*P* < 0.01.

The protein and mRNA expression of GFAP in the spinal cord of rats in the Model group was significantly upregulated when compared to that in the normal control group (*P* < 0.01, Figures 2(a) and 2(b)), indicating that the inflammatory injury of colon caused by acetic acid promoted activation of astrocytes in spinal cord. In contrast, expression of GFAP in the spinal cord in the EA group was significantly decreased when compared to the Model group (*P* < 0.01, Figures 2(a) and 2(b)), indicating that EA can inhibit the activity of astrocytes. Next, we checked the expression of P2Y_1_ receptor in the spinal cord. Compared to the normal control group, the expression of P2Y_1_ receptor in the Model group was significantly upregulated (*P* < 0.01, Figure 2(c)) similar to previous reports on the role of P2Y_1_ receptor in pathological pain [44], Moreover, the mRNA expression of P2Y_1_ in the Model group was also upregulated in comparison to that in the normal control group (*P* < 0.01, Figure 2(d)). Conversely, the protein and mRNA expression of P2Y_1_ in the spinal cord of rats in the EA group was significantly lower than that in the Model group (*P* < 0.01, Figures 2(c) and 2(d)), indicating that EA at Zusanli (ST 36) and Shangjuxu (ST 37) could inhibit the expression of P2Y_1_ in the spinal cord of rats with visceral hypersensitivity.

Further, we analyzed the levels of proinflammatory cytokines IL-6, IL-1*β*, and TNF-*α* in the spinal cord in response to EA by ELISA. We found that the level of IL-6 in the spinal cord of rats in the Model group was significantly upregulated when compared to the Normal group (*P* < 0.01, Figure 2(e)) suggesting that inflammatory cytokine IL-6 was involved in the process of spinal sensitization IBS visceral hypersensitivity induced by acetic acid which is in agreement with the previous reports on spinal sensitization of somatic pain [45]. However, the level of IL-6 in the spinal cord of rats in the EA group and in the FCA group was downregulated when compared to the Model group (*P* < 0.01, Figure 2(e)). Similarly, the level of IL-1*β* in the spinal cord of the Model group was significantly increased when compared to the Normal group but was found to be downregulated in both EA and FCA groups (*P* < 0.01, Figure 2(f)). Along the same lines, the concentration of TNF-*α* in the spinal cord of the Model group was significantly upregulated compared with the Normal group (*P* < 0.01, Figure 2(g)) and was downregulated in EA and FCA groups. These results suggest that EA inhibited the activity of astrocytes by reducing the release of IL-6, IL-1*β*, and TNF-*α* from astrocytes, so as to reduce the excitability of neurons related to the spinal dorsal horn and visceral nociceptive transmission leading to alleviation of visceral hypersensitivity.

### 3.3. Effect of ADP on Electroacupuncture-Mediated Regulation of PKC Pathway and MAPK/ERK1/2 Pathway

**Figure 3:**
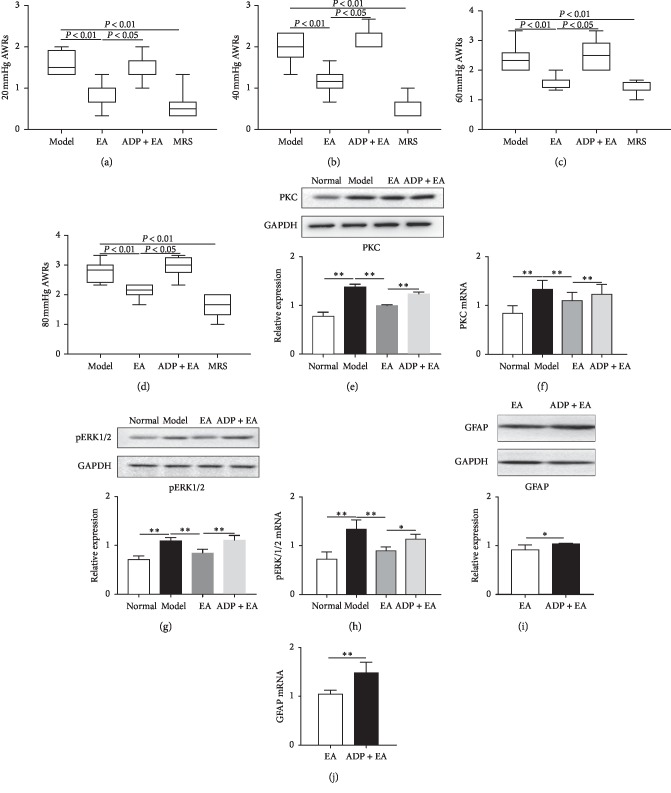
Effects of ADP on EA-mediated regulation of PKC pathway and MAPK/ERK1/2 pathway. (a)–(d) The effect of ADP on EA-mediated relief in AWR scores (20, 40, 60, and 80 mmHg CRD stimulation). (e) Expression of PKC protein in the L_6_-S_2_ segment dorsal spinal cord, *n* = 8. (f) Expression of PKC mRNA in the spinal cord, *n* = 8. (g) Expression of pERK1/2 protein in the dorsal spinal cord, *n* = 8. (h) ERK1/2 mRNA level in the dorsal spinal cord, *n* = 8. (i) GFAP protein in spinal dorsal, *n* = 8. (j) GFAP mRNA level in the dorsal spinal cord, *n* = 8.

Glial cells play an important role in chronic pain in addition to supporting and nourishing neurons. Activation of spinal glial cells and the release of inflammatory mediators and activation of various signaling pathways play an important role in pain modulation. To further explore the potential mechanism of the inhibitory effect of EA on the P2Y_1_ receptor in astrocytes, we analyzed the role of the P2Y_1_ receptor in the inhibitory action of EA on the activity of astrocytes by a behavioral method. We then tested the expression of PKC and pERK proteins that are activated by the P2Y_1_ receptor in astrocytes. As observed in Figure 3, compared to the Model group, AWR scores of rats in EA group were decreased: 20 mmHg (*P* < 0.05, Figure 3(a)), 40 mmHg (*P* < 0.01, Figure 3(b)), 60 mmHg (*P* < 0.01, Figure 3(c)), and 80 mmHg (*P* < 0.01, Figure 3(d)). However, the AWR scores of the ADP + EA group were higher than those in the EA group at 20 mmHg (*P* < 0.05, Figure 3(a)), 40 mmHg (*P* < 0.01, Figure 3(b)), 60 mmHg (*P* < 0.01, Figure 3(c)), and 80 mmHg (*P* < 0.01, Figure 3(d)). However, the AWR scores of the ADP + EA group were not statistically different compared with the Model group (*P* > 0.05, Figures 3(a)–3(d)). Compared with the Model group, the AWR score of the MRS group was significantly decreased: 20 mmHg (*P* < 0.01, Figure 3(a)), 40 mmHg (*P* < 0.01, Figure 3(b)), 60 mmHg (*P* < 0.01, Figure 3(c)), and 80 mmHg (*P* < 0.01, Figure 3(d)), suggesting that MRS, a P2Y_1_ receptor antagonist, inhibited visceral hypersensitivity suggesting that P2Y_1_ receptor plays an important role in the inhibitory regulation of EA on astrocytes.

The protein and mRNA expression of PKC in the Model group was higher than that in the Normal group (*P* < 0.01, Figures 3(e) and 3(f)), suggesting that the PKC signaling pathway was activated in chronic inflammatory visceral hypersensitivity. Compared to the Model group, the expression of PKC protein in the EA group was decreased (*P* < 0.01, Figure 3(e)), indicating that EA interfered with the upregulation of PKC protein by acetic acid in the spinal cord of rats with visceral hypersensitivity. However, the addition of the ADP (ADP + EA group) elevated the levels of PKC when compared to the EA group (*P* < 0.01, Figure 3(e)). These findings suggest that EA inhibits PKC activation downstream of the G-coupled P2Y receptor. We also analyzed the expression of pERK1/2 protein in the spinal cord which is also a downstream effector of the P2Y_1_ receptor. The protein and mRNA expression pERK1/2 in the Model group was upregulated when compared to Normal group (*P* < 0.01, Figures 3(g) and 3(h)), In contrast, EA downregulated the expression of pERK1/2 protein in comparison to the Model group (and this effect of EA was reversed by ADP (ADP + EA group) which showed a significant increase in pERK1/2 when compared to the EA group. Also, GFAP protein and mRNA level was significantly increased in the spinal cord of rats in the ADP + EA group compared with the EA group (*P* < 0.01, Figures 3(i) and 3(j)). Taken together, these findings suggest that EA inhibits the PKC pathway and MAPK/ERK1/2 pathways that are downstream of the P2Y_1_ receptor in the astrocytes.

### 3.4. Effect of ERK1/2 Inhibitor SCH on the Electroacupuncture-Mediated Inhibition of Astrocytes Activity

**Figure 4:**
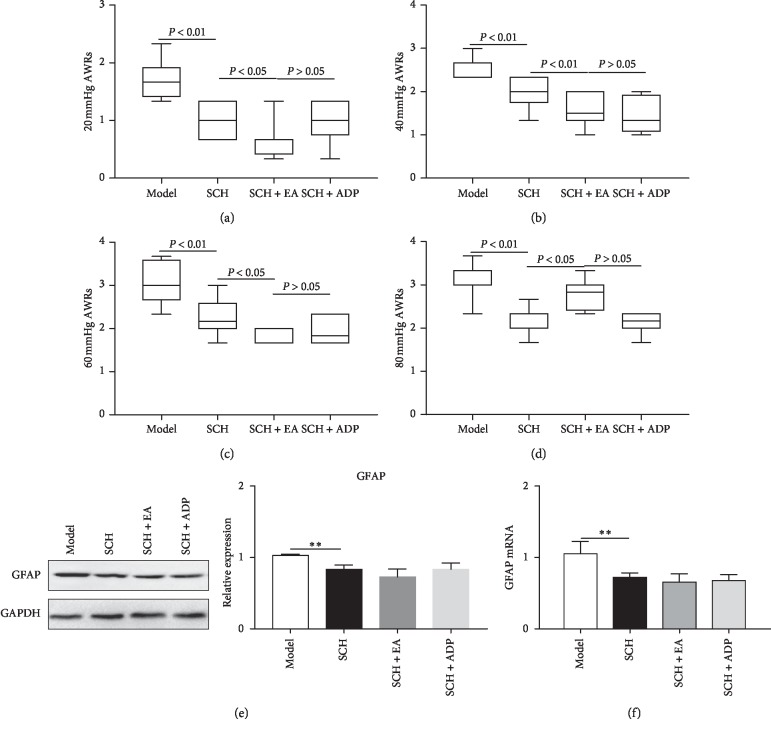
The effect of ERK1/2 inhibitor SCH on electroacupuncture-mediated inhibition of astrocyte activity. (a)–(d) AWRs scores under 20, 40, 60, and 80 mmHg CRD in the presence of ERK1/2 inhibitor SCH with and without EA. The experimental groups were Model, SCH, SCH + EA, and SCH + ADP. (e) GFAP protein level in the spinal cord, *n* = 8. (f) GFAP mRNA level in the spinal cord, *n* = 8.

ERK1/2 antagonist SCH [46] inhibits AWRs, suggesting the role of ERK1/2 in chronic visceral hypersensitivity. We treated the animals with SCH in the presence of absence of EA. We found that the AWR score of the SCH group was decreased when compared to the Model group in 20 mmHg (*P* < 0.01, Figure 4(a)), 40 mmHg (*P* < 0.01, Figure 4(b)), 60 mmHg (*P* < 0.01, Figure 4(c)), and 80 mmHg (*P* < 0.01, Figure 4(d)) CRD. The AWR scores of SCH + EA group rats were lower compared to the SCH group in 20 mmHg (*P* < 0.05, Figure 4(a)), 40 mmHg (*P* < 0.01, Figure 4(b)), 60 mmHg (*P* < 0.05, Figure 4(c)), and 80 mmHg (*P* > 0.05, Figure 4(d)) CRD, suggesting that ERK1/2 plays an important role in visceral hypersensitivity and is targeted by EA in relieving the pain. However, when compared to the SCH + EA group, there was no increase in the AWR score of the SCH + ADP group under the pressure of CRD stimulation (*P* > 0.05, Figures 4(a)–4(d)) indicate that the application of P2Y_1_ receptor agonist after intrathecal injection of ERK1/2 inhibitor SCH failed to increase the AWR scores. These findings suggest that ERK1/2 acts downstream of the P2Y_1_ receptor. We also examined GFAP expression in the spinal cord by Western Blot. GFAP in the SCH group was found to be significantly decreased when compared to the Model group (*P* < 0.05, Figure 4(e)), indicating that the intrathecal injection of ERK1/2 antagonist SCH inhibited the activity of astrocytes in the spinal cord of rats with chronic visceral hypersensitivity. GFAP level in SCH + EA group rats was even lower than that in the SCH group (*P* < 0.05, Figure 4(e)). However, no significant difference was observed between the expressions of GFAP in the SCH group and the SCH + ADP group (*P* > 0.05, Figure 4(e)), indicating that ERK1/2 inhibitor SCH blocked the upregulation of ADP on GFAP. The expression of spinal cord GFAP mRNA was consistent with the protein expression (Figure 4(f)). Taken together, these findings suggest that ERK1/2 plays an important role in the enhancement of astrocytes activity downstream of the P2Y_1_ receptor and this pathway is inhibited by EA.

## 4. Discussion

Visceral Hypersensitivity is the main pathological feature of IBS and acupuncture has a good advantage in regulating spinal cord sensitization. Acetic acid induced visceral hypersensitivity in normal rats which lasted till week 7. The behavior of experimental animals was found to be stable which is in line with the behavioral characteristics of visceral hypersensitivity in IBS. A large number of studies have reported the positive effect of EA in the treatment of IBS visceral hypersensitivity [47, 48]. In this study, we found that EA at Zusanli and Shangjuxu (100 Hz for 1.05 s, 2 Hz for 2.85 s alternately, pulse width for 0.1 ms, 1 mA, 30 min/d, once a day) significantly reduced the behavioral AWR score of IBS rats suggesting that EA significantly relieved the visceral hypersensitivity in our IBS rat. It is similar to the effect of EA on visceral hypersensitivity of IBS induced by acetic acid [36], colorectal distension neonatal rat [49], formaldehyde [50], and other injuries previously reported. Previous studies have shown that chemical inflammatory injury to the colon can cause peripheral and central sensitization and increased excitability in neurons [40] and that astrocytes play an important role in the occurrence and maintenance of sensitization [41]. Our findings that inhibition of astrocyte activity reduced visceral hypersensitivity of rats with IBS induced by acetic acid are consistent with the previously reported role of astrocytes in other types of inflammatory visceral hypersensitivity pathology [51]. Activated astrocytes release a large number of neuroactive substances, such as ATP, SP, prostaglandin, EAA, and CGRP, which act on presynaptic terminals and enhance the release of nociceptive neurotransmitters in primary afferent nerve endings and which also act on postsynaptic neurons to enhance their sensitivity and reactivity and increase the excitability of neurons [52]. In addition, synergistic effects among various proinflammatory cytokines form a positive feedback loop to promote central sensitization. Astrocytes release large amounts of proinflammatory cytokines such as IL-1*β* and TNF-*α* which promote the expression and phosphorylation of the AMPA receptor which plays a key role in LTP generation in the spinal cord dorsal horn [53]. Thus, inhibition of astrocyte activity plays an important role in inhibiting spinal sensitization.

EA can effectively inhibit visceral hypersensitivity induced by mechanical colorectal distention stimulation in adult rats [25]. We found that the inhibitory effect of EA at Zusanli (ST 36) and Shangjuxu (ST 37) on chronic visceral hypersensitivity induced by acetic acid was similar to that of FCA suggesting that EA may play a role in alleviating visceral hypersensitivity by inhibiting the activity of astrocytes. To further prove this hypothesis, we tested the effect of EA on protein and mRNA expression of GFAP as well as the levels of IL-6, IL-1*β*, and TNF-*α* in the spinal cord of rats with visceral hypersensitivity. Based on the behavioral results of the inhibitory effect of EA and FCA on IBS visceral hypersensitivity, it was verified that EA and FCA could relieve visceral hypersensitivity by regulating the content of inflammatory cytokines. Inflammatory injury to colon induced by acetic acid in the neonatal period could promote the activation of astrocytes in the spinal cord and maintain it until week 7. We have found that inhibition of this activation by intrathecal injection of FCA can inhibit the behavioral score of visceral hypersensitivity. EA can inhibit the expression of GFAP. We speculated that EA can relieve visceral hypersensitivity by inhibiting the activity of astrocytes. This will promote the clinical application of acupuncture to inhibit various kinds of spinal cord sensitization pain. Acupuncture is usually applied to deep tissue including muscle and connective tissue. ATP is released in large quantities in response to mechanical, electrical, and thermal stimulation. Once released, ATP acts as a transmitter that binds to various purinergic receptors, including P2X and P2Y receptors [36, 54, 55], completing the transformation process of acupuncture signal from mechanical stimulation to biological information [56]. The application of EA in lower limbs to regulate the function of internal organs is very interesting.

In this study, we also found that acetic acid intracolonic injection induced upregulation of P2Y_1_ receptor protein and its mRNA level in rat spinal cord which was inhibited by EA. In order to explore the intracellular mechanism of this inhibitory effect of EA, we analyzed the PKC signaling pathway downstream of the P2Y_1_ receptor in response to EA. ADP reversed the inhibitory effect of EA on visceral hypersensitivity. P2Y_1_ receptor was the target of the inhibitory effect of EA on the activity of astrocytes in the spinal dorsal horn of rats with IBS visceral hypersensitivity. As intracolonic injection of acetic acid activated the PKC protein in the spinal cord. EA inhibited PKC protein and mRNA in the spinal cord of rats with visceral hypersensitivity which is consistent with previous reports [46]. In our study, ADP reversed the inhibition of PKC by EA. Electrophysiological and molecular biological studies have found that ATP activates the P2Y receptor on the surface of neurons and glial cells. It is then coupled with different G proteins, induces the increase in [Ca^2+^]_*i*_ concentration, activates a variety of substrates including cAMP and PKC/MAPK, and triggers many signal transduction pathways [57, 58]. These findings suggest that EA inhibits PKC downstream of the P2Y_1_ receptor, which may further inhibit the increased intracellular Ca^2+^ in astrocytes, thereby blocking the activation of Gq in PKC signaling pathways. However, future studies are needed to test this hypothesis. The importance of extracellular-signal regulated kinase (ERK) in the mitotic signaling of P2YRs in astrocytes has been repeatedly confirmed [59] and MAPK/ERK has been shown to be involved in P2Y_1_ mediated proliferation of astrocytes [60, 61]. It has been demonstrated that astrocytes proliferate in vivo after being induced by intestinal inflammatory injury [62]. MAPK/ERK and PI3K/Akt pathways are also involved in the proliferation of astrocytes in vivo [63] and acute ERK1/2 activation is usually a cellular defense mechanism against injury [50, 64]. EA has been shown to modulate the MAPK/ERK pathway in the spinal cord to exert analgesic effects in somatic pain models [65, 66]. It is known that ERK1/2 acts downstream of the PKC signaling pathway and plays an important role in the pathogenesis of visceral hypersensitivity. Inhibiting PKC in the spinal cord can reduce behavioral scores in visceral hypersensitivity and inhibit ERK1/2 phosphorylation [67]. We found that acetic acid increased pERK1/2 protein and mRNA expression while EA reversed these changes. In contrast, ADP reversed the inhibitory effect of EA on pERK1/2 protein and mRNA expression.

**Figure 5:**
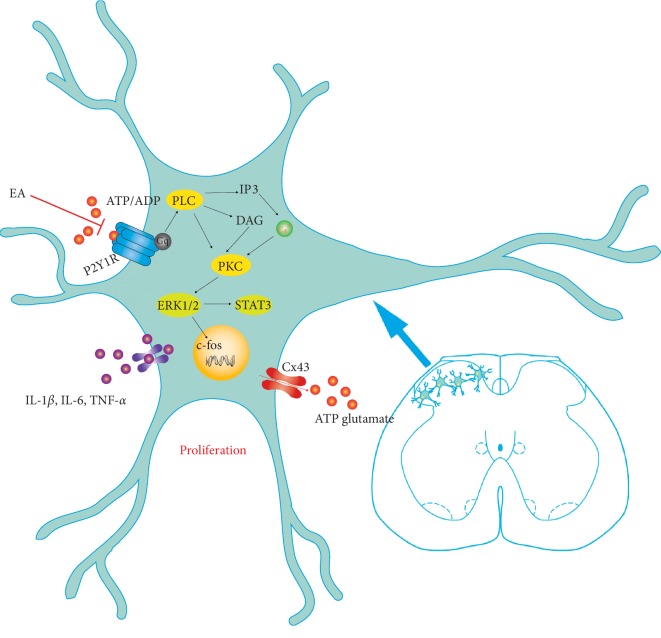
Schematic diagram of the mechanism of electroacupuncture in inhibiting the activity of astrocytes in the spinal dorsal horn.

In summary, EA inhibits the P2Y_1_ receptor on the surface of glial cells and the downstream protein kinase C and MAPK/ERK 1/2 pathway. In this study, acetic acid-induced colon inflammatory injury activated spinal cord astrocytes and PKC and ERK 1/2. EA can inhibit the activity of astrocytes by regulating the phosphorylation of PKC and ERK 1/2. The schematic diagram of the mechanism is shown in Figure 5. In view of the abovementioned role of the P2Y_1_ receptor in astrocytes activation, we speculated that the P2Y_1_ receptor mediates the regulation of astrocyte activity via PKC pathway and MAPk/ERK pathway and this pathway is inhibited by EA to alleviate visceral hypersensitivity and spinal cord sensitization. There are limitations to this study. We did not explore the mechanism of how EA inhibits the activation of the P2Y_1_ receptor. Furthermore, to explore the role of the MAPK/ERK pathway in astrocytes, not microglia, and neurons in inhibiting the activity of astrocytes by EA is the main research focus. Electrophysiological technology, as a real-time visual electrophysiological recording method in vivo, provides a possibility for us to deeply understand the peripheral and central neural mechanism of EA and provides cell-level evidence for the neuroscientific mechanism of EA.

## 5. Conclusion

EA inhibited astrocyte activity in the spinal cord dorsal horn of rat with IBS visceral hypersensitivity by inhibiting the P2Y_1_ receptor and its downstream, PKC, and MAPK/ERK1/2 pathways.

## Figures and Tables

**Table 1:** Abdominal withdrawal reflex (AWR) scores criteria.

No behavioral response to CRD stimulation	Score 0
Immobile during CRD and the occasional appearance of brief head motion after a pause at the onset of the stimulation	Score 1
A mild contraction of abdominal muscles, but no lifting of the abdomen of the platform	Score 2
A strong contraction of abdominal muscles and lifting of abdomen off the platform, no lifting of the pelvic structure of the platform	Score 3
Arching body and lifting of pelvic structure and scrotum	Score 3

## Data Availability

The data used to support the findings of this study are available from the corresponding author upon request.
